# Three-Dimensional Assessment of Temporomandibular Joint Using MRI-CBCT Image Registration

**DOI:** 10.1371/journal.pone.0169555

**Published:** 2017-01-17

**Authors:** Mohammed A. Q. Al-Saleh, Kumaradevan Punithakumar, Manuel Lagravere, Pierre Boulanger, Jacob L. Jaremko, Paul W. Major

**Affiliations:** 1 Department of Dentistry, Faculty of Medicine and Dentistry, University of Alberta, Edmonton, Canada; 2 Servier Virtual Cardiac Centre, University of Alberta, Edmonton, Alberta, Canada; 3 Department of Computing Science, University of Alberta, Edmonton, Alberta, Canada; 4 Department of Radiology and Diagnostic Imaging, Faculty of Science, University of Alberta, Edmonton, Alberta, Canada; Medical University of South Carolina, UNITED STATES

## Abstract

**Purpose:**

To introduce a new approach to reconstruct a 3D model of the TMJ using magnetic resonance imaging (MRI) and cone-beam computed tomography (CBCT) registered images, and to evaluate the intra-examiner reproducibility values of reconstructing the 3D models of the TMJ.

**Methods:**

MRI and CBCT images of five patients (10 TMJs) were obtained. Multiple MRIs and CBCT images were registered using a mutual information based algorithm. The articular disc, condylar head and glenoid fossa were segmented at two different occasions, at least one-week apart, by one investigator, and 3D models were reconstructed. Differences between the segmentation at two occasions were automatically measured using the surface contours (*Average Perpendicular Distance*) and the volume overlap (*Dice Similarity Index)* of the 3D models. Descriptive analysis of the changes at 2 occasions, including means and standard deviation (SD) were reported to describe the intra-examiner reproducibility.

**Results:**

The automatic segmentation of the condyle revealed maximum distance change of 1.9±0.93 mm, similarity index of 98% and root mean squared distance of 0.1±0.08 mm, and the glenoid fossa revealed maximum distance change of 2±0.52 mm, similarity index of 96% and root mean squared distance of 0.2±0.04 mm. The manual segmentation of the articular disc revealed maximum distance change of 3.6±0.32 mm, similarity index of 80% and root mean squared distance of 0.3±0.1 mm.

**Conclusion:**

The MRI-CBCT registration provides a reliable tool to reconstruct 3D models of the TMJ’s soft and hard tissues, allows quantification of the articular disc morphology and position changes with associated differences of the condylar head and glenoid fossa, and facilitates measuring tissue changes over time.

## Introduction

TMJ internal derangement represents abnormal changes of the articular disc position in relation to the mandibular condyle and temporal bone glenoid fossa. The articular disc is a dense fibro-cartilaginous tissue that interposes between the articular surfaces of the TMJ. The articular disc allows smooth movement of the incongruent surfaces of the mandibular condyle, glenoid fossa, and articular eminence. Also, it dissipates the compression forces transmitted during the joint function. The changes in disc position alter the dynamic forces inside the joint, which stimulate an adaptive response that involves altered osseous contour. The association between TMJ internal derangement and changes in joint space and osseous contour has been established in the literature. [1–3]

Adequate diagnosis is essential for correct treatment. In addition to the observer’s expertise, clear image information is a substantial factor that leads to correct diagnosis. The field of image processing has been very active in the past decade, and the recent advancements in 3D volume rendering of human anatomical tissues allowed better visualisation and assessment of the tissues morphology and dynamics. The TMJ articular disc derangement is a 3D problem that is commonly described and diagnosed from two-dimensional (2D) images. In addition, 3D models of the TMJ enable quantitative analysis of tissue changes in all directions. Multiple attempts have been conducted to visualise the TMJ articular disc and osseous structures in 3D using MRI.[4–13] Unfortunately, differentiation of osseous contours from MRI is often insufficiently clear, especially in the TMJ region.[14]

The TMJ cartilaginous disc is best depicted on MRI and osseous surfaces are best seen in CT. The cone beam CT (CBCT) has a substantial lower radiation dose when compared to helical CT and has become the predominant CT approach in the fields of dentistry, maxillofacial orthognathic surgeries, and TMJ assessment. Fusing MRI and CBCT imaging have been recently introduced to assess TMJ pathology.[15] MRI and CBCT images are registered in a common spatial 3D coordinate system first before their final fusion. The fused images provide a desirable complementary information of the articular disc and osseous surfaces for optimum diagnosis. The registration process to generate MRI-CBCT images has been shown to be accurate and reliable in TMJ internal derangement assessment.[16,17]

In this study, we had two objectives: 1. to describe a new approach to construct a 3D model of the TMJ using MRI-CBCT registered images; and 2. to evaluate the intra-examiner reproducibility values of reconstructing the 3D models of the TMJ.

## Materials and Methods

### Patients

Five adult patients with no history of TMJ dysfunction, undergoing investigation for possible oral squamous cell carcinoma were recruited from the Division of Otolaryngology Head and Neck Department at the University of Alberta. Patients had MRI and CBCT imaging for assessment of TMJ abnormality before going for surgery. The study was approved by the Human Research Ethics Board at the University of Alberta. Patients were provided with detailed explanation of the study and they provided their written formal consent to participate in the study. Images were obtained at closed mouth with maximum inter-cuspation position using centric occlusion bite stent made of polyvinylsiloxane material.

### MRI acquisition

The MR images were obtained in the supine position with a multi-channel 12-element head array coil in 1.5 Tesla scanner (Siemens Syngo MRB17, Erlangen, Germany), without sedation or intravenous contrast agent administration. Four MRI weighted sequences were obtained: Mouth-closed oblique sagittal Proton Density (PD)-weighted images with a small FOV 13cm x13cm, a slice thickness of 3 mm (14 slices per TMJ), an inter-slice gap spacing of 0.3 mm, an echo time 11msec and a repetition time 1800 msec. Mouth-Closed oblique sagittal T2-weighted spoiled gradient echo 3D sequence was obtained with a FOV of 14 cm x 12 cm, a slice thickness of 3 mm, an echo time of 95 msec and a repetition time of 36.3 sec and a voxel size of 0.8 x 0.5x 3 mm^3^.

Mouth-closed coronal sagittal PD-weighted images was obtained with a small FOV of 19 cm x 9.5 cm, a slice thickness of 2 mm (16 slices per TMJ), an inter-slice gap of 2 mm, an echo time of 14 msec and a repetition time of 1800 msec. Mouth-open oblique sagittal PD-weighted images with a FOV of 12 cm x 12 cm, a slice thickness of 3 mm, an inter-slice gap of 0.3 mm, a repetition time of 1800 msec, an echo time of 15 msec and a voxel size of 0.6 x 0.5 x 3.0 mm^3^.

### CBCT image acquisition

CBCT images were acquired to capture maxilla, mandible and both TMJ condyles. Scans were obtained in 360 degrees of rotation with Frankfort plane parallel to the floor and making sure the subject was in upright position. Scan was obtained for a medium FOV of 16 cm x 13cm, a voxel size of 0.25 mm^3^ at 26 seconds scan time (120KVp, 5mA).

### Image-registration using mutual information

**Fig 1:**
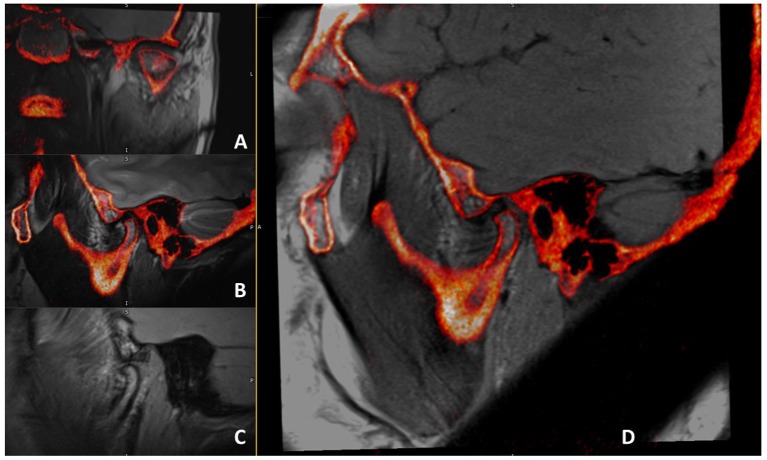
Multiple MRI sequences and CBCT images after registration. A: Oblique coronal PD-weighted MRI-CBCT registered image; B: Oblique sagittal T2-weighted MRI-CBCT registered image; C: Open mouth oblique sagittal PD-weighted MRI only. D: Oblique sagittal PD-weighted MRI-CBCT registered image.

**Fig 2:**
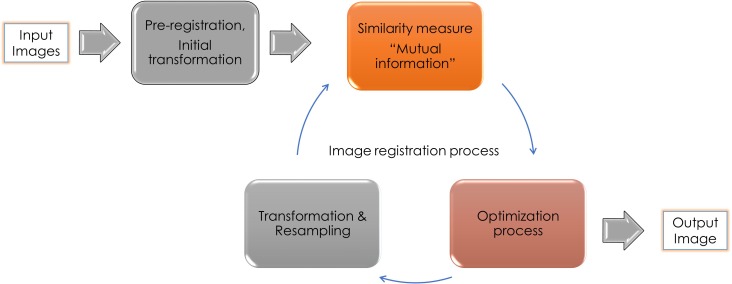
Sequence of the different automated image processing steps from the set of two input images to the final fused output image.

Digital Imaging and Communication in Medicine (DICOM) files of all images were transferred to co-registration processing software to perform multi-modality image registration for the MRI sequences and CBCT data sets. All images were automatically registered to a common 3D Cartesian coordinate system (x, y, z) and fused into a common volume for assessment (Fig 1). The multi-modality image registration process involves a combination of three processes; computation of similarity measure, optimization algorithm, and space transformation (Fig 2).

#### 1.Similarity measure

A rough initial 3D alignment of images is necessary, which can be done automatically by considering similarity of images. Among many similarity measure algorithms, normalized mutual information is considered the most common and reliable algorithm for multi-modal image registration.[18–21]

**Fig 3:**
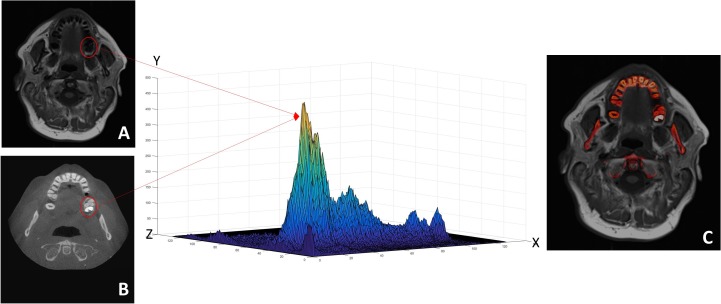
**Illustration shows a joint histogram of 2 successfully registered multimodal images (A: MRI and B: CBCT) using normalized maximum mutual information approach.** The Y-axis in the histogram represents the voxels intensities’ distribution from MRI and CT, Z-axis represents the voxels’ values of the MRI and X-axis represents the voxel’s values of the CBCT image. The radio-opaque (bright) molar tooth in the CBCT has similar intensity distribution to the low intensity (dark) region in the MRI, therefore, voxels from both images were matched and correspond to each other in the histogram. The finally fused registered image using this technique is displayed in Fig 3-C.

The grey-level intensity values from MRI and CBCT images do not linearly correspond. Therefore, a similarity measure function that utilize the statistical dependence of the voxels intensities’ distribution was used, called “*normalized mutual information*”. The normalization process starts with a joint entropy histogram to measure the similarity of intensities’ distribution in both images, and the voxels’ clusters of MRI and CBCT images are then matched accordingly. The joint histogram appears the sharpest when the two images are completely and perfectly aligned. In the case of images from different modalities, the images’ intensities are entirely different, and finding a sharp joint histogram is not easily attainable. As seen in Fig 3, the highest attenuating or brightest points on CBCT scan (molar tooth enamel) corresponds to a very low signal on MRI.

Mutual information is less sensitive to the inherent noise of MRI and CBCT images compared to other common measures such as sum of squared differences.[18] The mutual information based similarity measure between two voxels *X* and *Y* is defined as:

$$MI\;(X,Y)=\sum_{y\in Y}\sum_{x\in X}p(x,y)\text{log}(\frac{p(x,y)}{p(x)p(y)})$$

where *p* (*x*,*y*) is the joint probability function. The corresponding voxel values in the CBCT (*U*) and the transformed MRI volume (*V*) are *X* and *Y* respectively. The function *p*(*y*) is the marginal probability of the intensity *X* appearing in volume *U*; and the function *p*(*y*) is the marginal probability of the intensity *Y* appearing in transformed volume *V*. Intuitively, mutual information measures the information that *X* and *Y* share as it measures how much knowing one of these variables reduces uncertainty about the other.

#### 2.Optimization algorithm

The optimization process attempts to estimate the transformation that yields the highest similarity between the voxels clusters in the joint entropy histogram. This process is more complex in multimodal images since similarity is not easy to define. This is an iterative process that optimizes the similarity measure of one criterion at a time, until no criterion or similarity measure are changing any longer. The above mutual information problem can be solved using Powell’s conjugate direction method of optimization, which starts with fast rough optimization followed by an accurate slow one.[18,20]

#### 3.Spatial transformation

**Fig 4:**
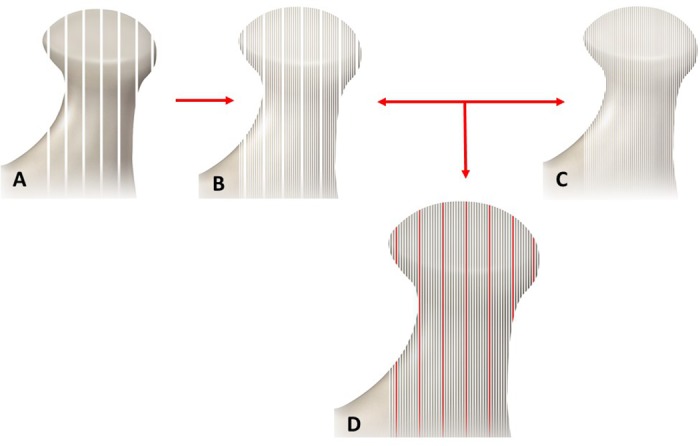
Illustration shows lateral (sagittal) view of the TMJ to explain the resampling process of 2 images of the TMJ. A: An MRI for the TMJ with 8 sagittal sections each with 3mm thickness and inter-section gap of 0.3mm. B: Resampled sagittal sections of the MRI to match section thickness of CBCT image. C: CBCT image with 0.3mm of section thickness. D: Merged MRI and CBCT images with similar section thickness. The red lines represent the intersection gap from the MRI that is filled with a repeated adjacent image section. The resampling process allows for the computation of mutual information for MRI and CBCT images with different resolutions.

Optimization progresses with subsequent image transformation and similarity measure to align two image volumes in a rigid/linear fashion (scale, translation and rotation only). The transformation matrix $\hat{T}$ is obtained using the registration algorithm to reconstruct the original image volumes. A resampling of one image (usually the image with the lowest spatial resolution to maintain image quality) is required to form a joint image histogram and compute similarity metric. An example of resampling an MRI and CBCT image set is illustrated in Fig 4. During the proposed registration process, the CBCT volume *U* is considered fixed and the MRI volume *V* is moving with transformation *T*. We state the problem of aligning image volumes as the optimization of a similarity measure based on mutual information:

$$\hat{T}=\underset{T}{\mathrm{arg max}}\;MI\;(U,V,T)$$

where *MI* is the mutual information between volumes *U* and *V* after transformation *T*.

### Segmentation and 3D rendering of the disc, condyle and fossa

**Fig 5:**
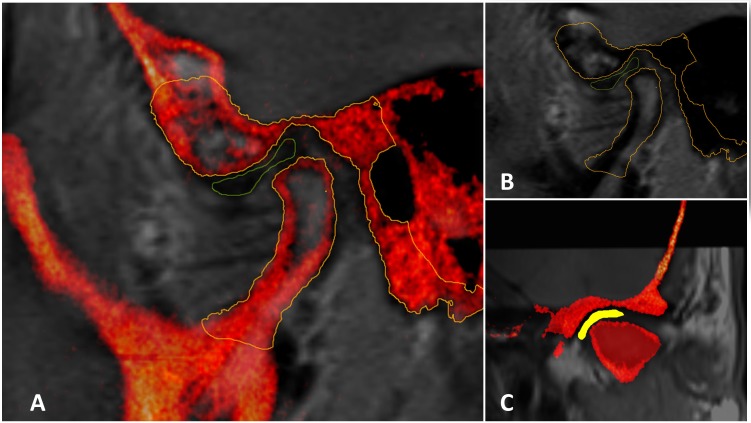
Tissues segmentation. A: Oblique sagittal PD-weighted MRI(gray)-CBCT(red) registered image showing outlined/segmented articular disc and condylar head and TMJ structure of the temporal bone. B: Oblique sagittal PD-weighted MRI only showing the outlined/segmented osseous structures from the co-registered CBCT. C: Oblique coronal PD-weighted MRI-CBCT registered image showing the same outlined/segmented structures.

**Fig 6:**
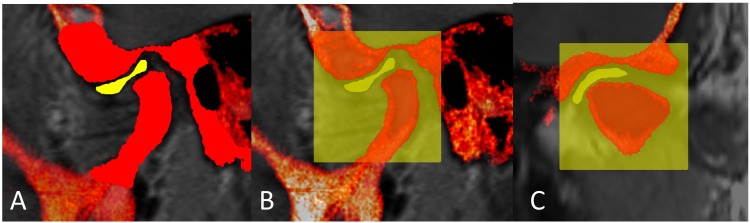
Tissues segmentation in MRI(gray)-CBCT(red) registered images. A: Oblique sagittal PD-weighted MRI-CBCT registered image showing outlined/segmented articular disc (yellow) and condylar head and TMJ structure of the temporal bone (bright red). B: A yellow 3D cropping box of about 2.5cm^3^ in dimensions was manually drawn to export the cropped TMJ structures only as STL files. C: Oblique coronal PD-weighted MRI-CBCT registered image showing the medio-lateral dimensions of the same cropping box highlighting the cropped TMJ structures.

Using Mirada XD software, the gray-value threshold of the condylar head and glenoid fossa on each sagittal section in the entire region of interest (~80 sections, of 0.25mm thickness) was automatically highlighted. The gray-value threshold represents pixel intensity of the osseous structures in the CBCT images were roughly ranged between 300–1000 Hounsfield units based on the quality of the scan and the location of the section. By adding or erasing, the outlined structures were manually corrected by the first author to obtain accurate segmentation, and therefore, the process can be considered a semi-automatic segmentation (Fig 5). Once the condylar head and glenoid fossa were defined, a cropping box of about 2.5cm^3^ in dimensions was manually drawn to include the condylar head and glenoid fossa the posterior slope of the articular eminence (Fig 6). The pitch, roll and yaw values of the cropping box were saved and used again to crop the same tissues in the second time of segmentation. Finally, the delineated tissues were exported as 3D models in STereoLithography (STL) format using Scan IP software (Simpleware, Exeter, United Kingdom).

**Fig 7:**
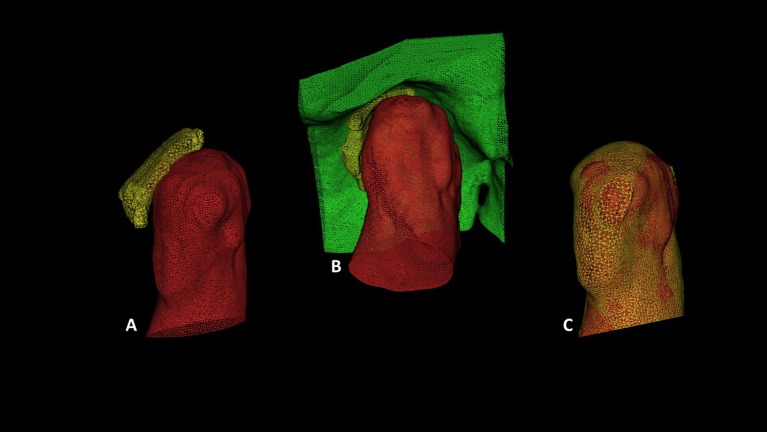
Illustration shows lateral (sagittal) view of the TMJ in 3D models rendered using STL files. A: shows the condylar head (red) and the articular disc (yellow). B: shows the condylar head (red); glenoid fossa (green) and articular disc in between the 2 structures (yellow); C: shows overlapped condyles from two trials of segmentation by the same reader.

The defined osseous structures of the TMJ outlined the joint space in the MRI-CBCT registered image. The first author, a TMJ disorders’ specialist with 4 years’ experience in TMJ MR diagnostic imaging, manually traced the voxels comprising the articular disc in all sections of the MRI. The manual segmentation took about 20–30 minutes for each disc. The articular disc is depicted by low signal intensity in all PD-weighted and T2-weighted images. The PD-weighted coronal sections were checked for further editing as well. Once the articular disc was finally defined, the 3D model was constructed and saved as using the STL format (Fig 7A and 7B). To compute accurate values for the intra-observer variability, we abstained from applying any smoothing algorithms in generating the 3D segmented surfaces and retained the original user-defined manual contours.

### Reproducibility assessment of tissue segmentation

The 10 TMJs (articular disc, condyle and glenoid fossa) were segmented at two different occasions, with at least one-week apart, by the same reader to assess intra-examiner reproducibility (Fig 5C). Differences were automatically measured using the *average perpendicular distance* of the models’ surface contours and the volume overlap (Dice Similarity Index).

#### 1. Average perpendicular distance

The perpendicular distances between all corresponding surface contour points of the time 1 and time 2 models were measured and the root mean squared distance (RMSD) and maximum distance (MD) were detected. The higher the value of the *SMD and MD* the greater the mismatch between the 2 models.[22]

#### 2. Dice similarity index (DSI)

The volume overlap of the two models was measured using the following:

$$DSI(M_{1},M_{2})=\frac{2M_{1}⋂M_{2}}{\left(M_{1}+M_{2}\right)}$$

Where *M*_*1*,_ *M*_*2*_ *and M*_1_ ⋂ *M*_2_ are the volumes of the time 1 model, time 2 model and the intersection between them, respectively. The *DSI* value is set between 0–1, where 1 means perfect match.[23]

### Statistical analysis

Descriptive analysis of the changes between the 3D models from 2 occasions, including means and standard deviation (SD) were computed to evaluate the intra-examiner reproducibility in reconstructing the TMJ 3D models.

## Results

Table 1 shows the descriptive analysis of the measured data. The source data (volume and all dimensions) are reported in S1 Table. The condyle 3D models showed the lowest change between the 2 times of segmentations (RMSD = 0.1±0.08; MD = 1.9 ±0.93 & DSI = 0.98 ±0.02), followed by the glenoid fossa 3D models (RMSD = 0.22 ±0.04; MD = 2 ±0.52 & DSI = 0.96 ±0.03), then the articular disc (RMSD = 0.3 ±0.1; MD = 3.6 ±0.32 & DSI = 0.80 ±0.1).

**Table 1:** Intra-observer variability in measurement of the surface contour changes and Dice Similarity Index for mandibular condyle, glenoid fossa and articular disc. (RMSD: Root mean squared distance; MD: Maximum distance; DSI: Dice similarity index.**)**

	Condyle			Glenoid fossa			Articular disc		
TMJ	RMSD (mm)	MD (mm)	DSI	RMSD (mm)	MD (mm)	DSI	RMSD (mm)	MD (mm)	DSI
1	0.18	2.92	0.97	0.24	1.42	0.97	0.26	3.92	0.88
2	0.28	0.87	0.94	0.28	1.86	0.93	0.27	2.97	0.89
3	0.1	2.60	0.99	0.19	2.1	0.99	0.36	3.95	0.78
4	0.08	2.36	0.99	0.17	1.9	0.96	0.33	3.84	0.75
5	0.05	1.45	1	0.20	1.81	0.99	0.41	3.81	0.86
6	0.09	2.79	1	0.21	2.27	0.99	0.29	3.5	0.76
7	0.05	1.45	0.93	0.20	1.81	0.97	0.41	3.81	0.80
8	0.09	2.79	1	0.20	2.27	0.99	0.29	3.5	0.73
9	0.09	2.02	0.98	0.28	1.43	0.90	0.23	3.2	0.71
10	0.02	0.15	1	0.27	3.26	0.99	0.29	3.7	0.90
Mean (SD)	0.1 (0.08)	1.9 (0.93)	0.98 (0.02)	0.22 (0.04)	2 (0.52)	0.96 (0.03)	0.3 (0.1)	3.6 (0.32)	0.80 (0.1)

## Discussion

MRI has been considered as the standard, non-invasive, diagnostic imaging tool for patients with clinical symptoms of TMJ soft tissues pathology.[24–28] However, TMJ osseous structures are not well depicted in these routine MRI.[14,29] Since the articular disc position is evaluated according to its relationship with articular condyle and eminence,[24] poor contrast between the articular disc and outlining cortex of the condyle and posterior slope of the eminence make image interpretation a difficult task. Observer variation in detecting disc position in MRI has been cause of concern even among experienced radiologists.[24,30–33] Use of a MRI-CBCT registered image not only allows assessment of the articular disc shape and position, but also allows for analyzing the condyle shape and location. Fused MRI-CBCT image facilitates accurate 3D reconstruction of the articular disc, condylar head and articular eminence, and allows multi-dimensional quantification of the TMJ changes. Here, we explained and demonstrated the reliability of the process of image fusion and 3D reconstruction for TMJ.

### Registration of multiple MRIs

During function, the normal articular disc interposes between the condylar head and articular eminence and moves antero-posteriorly. Internal disc derangement often includes medio-lateral and rotational displacements beside anterior displacement. The articular disc consists of dense fibrous collagenous connective tissue. It has low signal intensity and appears as void or dark biconcave structure in different MRI sequences. Muscle fibers in the lateral pterygoid muscle and the highly vascular retrodiscal tissues appear with higher signal intensity than the articular disc. Studies in the literature have used multiple MR protocols, include different acquisition planes, weighting sequences, repetition time, echo time and slice thicknesses, to image the TMJ. The MRI PD-weighted image is considered the best sequence to visualize the TMJ anatomy.[4,5,10,34] Occasionally, the magic-angle phenomenon is encountered where the posterior band of the disc has high signal intensity and is confused with the highly vascularized retrodiscal tissues at the PD-weighted or T1-weighted sequences. Increasing the time of echo, as applied in T2-weighted sequence, exposes the magic angle phenomenon and prevents false-positive diagnosis of shortness in disc length or anterior disc displacement. Additionally, T2-weighted sequences add a clinical value in diagnosing inflammation in TMJ capsule, bone marrow edema and joint effusion around the articular disc.[34,35]

Reducing the slice thickness requires longer scanning time and increases the motion artifact chances. The inter-slice gaps are necessary to prevent the cross-talk artifact and poor signal to noise ratio, however, large gaps result in missing parts of the already small disc. To prevent interferences between MRI slices and reduce cross-talk artifact, inter-slice spacing is placed and to varies from 10–20% of the slice thickness in different imaging protocols. Imaging specifications suitable for routine clinical examination are challenging when used for articular disc 3D reconstruction. In our study, minimal inter-slice thickness was placed 0.3mm (10%) to reduce missing special anatomical information. Only minute deformities may be missed in these images. Registering multiple MRI sequences to CBCT image with unified x,y, and z coordinates improves disc morphology visualization,[16] and potentially improves the disc segmentation accuracy and reduce the operator error. The accuracy of the mutual-information-based MRI-CBCT registration protocol was measured in a previous study by the same authors of the present study. [17] The authors used screw-mounted fiducial markers in cadaver swine heads to measure the linear target errors of the image registration technique. The detected target was error was 0.2±1.2mm. [17]

### Segmentation and 3D volume rendering of the articular disc

MRI-CBCT registration as performed here uses routine imaging protocols widely performed in dentistry and TMD clinical practice. The overlapped CBCT image sharply outlined the condyle and articular eminence with clear strong contrast between osseous structure and articular disc in MRI. Attempts to depict the TMJ internal structures in 3D have been reported to better understand the cause and effect relationship between TMJ changes and dysfunction.[4,5,8–13] The 3D imaging quantifies the relationships and describes dynamics between the joint structures. 1992, Price *et al*,[9] made the first attempt to build a TMJ 3D model by digitizing manually-traced sagittal and coronal MRI slices. The tracings were imported as series of projections to form a 3D wireframe and the authors reported range of error between 0.5–3.2mm.[9] Digitizing the manually traced images adds an additional unquantifiable error to the segmentation process. Motoyoshi *et al*.[8] reconstructed the TMJ model from 2D multi-slice T1-weighted MR images, using image processing software (Microsoft Visual Basic). The darkest gray pixels where automatically selected to depict the articular disc. Although manual tracing of 2D slices was avoided in their study, the fully automatic detection of the articular disc in MRI using pixels’ value was not clearly explained. Similarly, Smirg *et al*.[13] assumed that the darkest voxels’ clusters between the condyle and the glenoid fossa likely belonged to the articular disc, which was automatically segmented without separating the other surrounding tissues. Chirani *et al*.[10] reduced the slice thickness of the MRI to 2mm, which is below clinical standard (3mm), and applied image enhancement filter to minimize the residual noise due to narrow slice thickness. Image enhancement added a potential error factor to the boundaries of the targeted object and may have led to underestimate or overestimate the boundaries of the low intensity articular disc. Hayakawa *et al*.[5] and Kober *et al*.[4] outlined the whole space between the condyle and the glenoid fossa/articular eminence, including the lateral pterygoid muscle from a PD-weighted sequence. The outlined area was processed in color scale to visualize the low intensity articular disc. Mikulka *et al*.[11,12] introduced automatic technique to segment the articular disc based on edge analysis in addition to the statistical analysis of the region (active contouring). The technique extracts the disc region between the condyle and the glenoid fossa, and subdivide it into sub-regions with different mean intensities. Median noise filtering was used, for images with low signal-to-noise ratio and poorly defined edges, to reduce noise without blurring the edges of the assumed disc region. These studies had two major limitations; First, the assumption that the articular disc lies always within the glenoid fossa; Second, dependency on the poorly outlined surrounding osseous structures that have low MRI signal intensity and may have been included in the segmentation process. It was not clear how was it possible for the authors to define the articular disc out of the other surrounding soft tissues, especially when the articular disc can be easily confused with the surrounded tissues such as the lateral pterygoid muscle tendon and the cortex layer of the condyle and articular eminence. The reported automatic techniques require high image quality with sharp resolution and contrast to clearly distinguish the articular disc from the surrounding soft and hard tissues.

The current studies in the literature mainly focus on segmenting the articular disc automatically within a reasonable time, and with the least possible level of operator interaction. Automatic segmentation is best applied when a clear difference in intensity between foreground region and background region is detected. In small tissues, such as articular disc that is represented by small number of voxels and lie within surrounding tissues with similar signal intensity at low signal-to-noise ratio MRI, manual or semi-automatic segmentation with experienced operator interaction is likely a more reliable technique to accurately detect the disc. We found manual segmentation was acceptable between attempts by the same operator, with maximum distance change in articular disc of 3.6±0.32mm, similarity index of 80% and root mean squared distance of 0.3±0.1mm. Unfortunately, there is no study in the literature reporting reproducibility or reliability of a manual disc segmentation to be compared with our findings.

### Segmentation and 3D volume rendering of the TMJ osseous structures

All studies in the literature that obtained MRI to visualize TMJ, have utilized less than optimal images to outline and segment osseous structures in 3D.[4,5,8–13] CT and CBCT remain the gold standard for osseous pathology diagnosis especially flattening, osteophyte and increased joint spaces of the TMJ. MRI cannot sufficiently differentiate osseous structures for 3D segmentation due to its inherent limitations (i.e. large slice thickness, inter-slice gaps, cross-talk artifact, and high signal-to-noise ratio). Hayakawa *et al*.[5] and Kober *et al*.[4] segmented the condyle semi-automatically from 3mm slice thickness with 20% inter-slice space, after filtering the condyle contour. The large gap between slices can result in deficient reconstruction of a 3D condyle. In addition, filtering was applied to reduce noise and reconstruction artifacts; however, the resulted blurred edges can lead to overestimated or larger region than the original structure. Smirg *et al*.[13] used the marker-controlled watershed algorithm to outline the condyle, by separate areas with high signal intensity from the surrounding tissues. The algorithm is very sensitive to signal-noise ratio, which renders it unsuitable for the routine TMJ MRI. Schilling *et al*.[36] reported the reliability of superimposing two condylar heads’ 3D models reconstructed from two TMJ CBCT images obtained at two occasions. The images had 0.5mm voxels size and were co-registered using a best-match technique. Using semi-automatic segmentation, the authors reported inter-observer mean difference ranged between 0.4–0.6mm with excellent reliability (Interclass coefficient >0.75). The reported values were similar to the values in this study similarity index of 98% and root mean squared distance of 0.1±0.08mm).[36] The difference between the two studies may be attributed to the difference in voxels’ size and registration technique. Bone segmentation reliability is more dependent on the intensity threshold that varies by different machines and software, and less dependent on the operator experience and/or judgement.

### Limitations and future recommendations

1. Patient motion during imaging remains an inherent error source. Patients should be asked to remain immobile during scanning, and heads can be stabilized by being fastened into a special head holder. Occlusal splints in the maximum inter-cuspation position are necessary to guarantee the condylar position in both MRI and CBCT images. Other MRI inherent artifacts such as metallic susceptibility (dental work, vascular clips), chemical shift, aliasing, truncation and pounce point artifact should be considered as well.
2. Although, the MRI-CBCT image registration in open mouth is technically possible, additional open-mouth CBCT images may not be necessary since they don’t provide additional diagnostic information. Images were taken in close-mouth position to standardize the measurements of the disc changes.
3. The manual segmentation of the disc by an experienced operator seems to be the most reliable approach, however, it’s a tedious process and highly operator dependent. Operator fatigue, low experience and repeatability are all potential error sources.
  Although the TMJ MRI protocol used in this study is the most clinically relevant protocol, the low number of the MRI sections can be a source of segmentation error. Three dimensional MRI acquisition of TMJ or 2D acquisition with small slice thickness can be considered in the future.
4. Enhanced image resolution with 3T MRI with dedicated TMJ coil may improve segmentation accuracy. However, 1.5T is often used in clinical practice due to accessibility and cost. By using 1.5T we demonstrated a clinical relevant approach. Future studies comparing segmentation between 1.5T and 3T images would be useful.
5. The measured differences in the structures’ segmentation were subject to an inevitable software quantization and the choice of points (the software truncates the numerical contour location values to the nearest pixel location) potential errors.

Although the proposed method is, somewhat, time consuming and requires operator interaction, it’s the first method that incorporates TMJ structure from two imaging sources. Also it allowed outlining the articular disc from multiple overlapped MRI sequences. The mutual information multimodal image co-registration has substantial potential for further exploration in this field. Further research shall be continued to improve the time factor and the operator dependency.

## Conclusion

This study presented a new approach to simultaneously visualize the TMJ osseous and soft tissue structures in 3D, from a multiple MRI sequence images that were spatially registered with CBCT image. The MRI-CBCT registration provides a reliable tool to reconstruct 3D models of the TMJ’s soft and hard tissues and allows quantification of the articular disc morphology and position changes with associated differences of the condylar head and glenoid fossa. The reconstructed 3D models are quantifiable in terms of volume and x-y-z linear measurements, which facilitate measuring tissue changes over time. The MRI-CBCT image registration has a potential to be used in other research and clinical applications.

## Supporting Information

S1 Table The source data, volume and all dimensions, for mandibular condyle, glenoid fossa and articular disc at time 1 (T1) and time 2 (T2). (DOCX)
