# Supplementary material for: Three-Dimensional Assessment of Temporomandibular Joint Using MRI-CBCT Image Registration
Source: PLoS One. 2017 Jan 17;12(1):e0169555. doi: 10.1371/journal.pone.0169555 (PMC5241008; doi:10.1371/journal.pone.0169555)
Supplement: S1 Table — (DOCX) [file pone.0169555.s001.docx]

| ***TMJ*** | | ***Condyle*** | | ***Glenoid Fossa*** | | ***Articular disc*** | |
| --- | --- | --- | --- | --- | --- | --- | --- |
|  |  | ***Volume*** | ***(x,y,z)*** | ***Volume*** | ***(x,y,z)*** | ***Volume*** | ***(x,y,z)*** |
| 1 | *T1* | 1.43 | (18,14.5,16.7) | 1.71 | (24.7,19.5,10.2) | 0.28 | (16.3,14.2,9.7) |
|  | *T2* | 1.47 | (17.7,15,17.2) | 1.78 | (24.5,19.0,10.5) | 0.25 | (16.2,11.5,10) |
| 2 | *T1* | 1.56 | (19.7,13,16.2) | 1.62 | (25.0,19.5,11.0) | 0.12 | (16.2.8.7,9.0) |
|  | *T2* | 1.62 | (20.5,11.7,17) | 1.80 | (25.1,19.0,11.2) | 0.11 | (15.3,8.5,8.7) |
| 3 | *T1* | 1.46 | (18.5,18.7,15.2) | 2.10 | (19.5,22.0,9.5) | 0.14 | (16.7,11.2,9.0) |
|  | *T2* | 1.39 | (17.5,18.5,14.2) | 2.10 | (19.7,22.5,9.1) | 0.16 | (16,11.75,9.3) |
| 4 | *T1* | 1.72 | (18.7,19,15) | 2.13 | (25.0,19.2,13.5) | 0.09 | (13.3,8.0,7.7) |
|  | *T2* | 1.82 | (19.7,19,16) | 2.30 | (25.1,18.9,13.5) | 0.10 | (13.5,8.3,8.2) |
| 5 | *T1* | 1.61 | (22.7,18,16.5) | 1.53 | (23.0,19.7,14.2) | 0.08 | (11.7,6.0,3.7) |
|  | *T2* | 1.71 | (21.7,17,16.7) | 1.54 | (23.1,19.5,14.5) | 0.07 | (9.5,5.5,6.5) |
| 6 | *T1* | 1.52 | (19.2,13.2,16.7) | 1.46 | (24.0,17.5,11.7) | 0.09 | (11.5,5.0,7.2) |
|  | *T2* | 1.45 | (18.2,14.2,15.5) | 1.50 | (24.1,17.7,11.5) | 0.07 | (10.5,5.5,3.7) |
| 7 | *T1* | 0.78 | (13.5,15,11.5) | 1.31 | (16.2,22.5,12.5) | 0.03 | (5.3,4.7,3.0) |
|  | *T2* | 0.68 | (12.3,15,10.9) | 1.30 | (16.1,22.4,12.4) | 0.02 | (5.5,6.0,2.0) |
| 8 | *T1* | 0.59 | (13.7,14.5,11.2) | 1.37 | (15.6,19.7,9.0) | 0.01 | (5.3,5.8,2.0) |
|  | *T2* | 0.62 | (13.5,15.5,12.2) | 1.36 | (15.7,19.5,9.0) | 0.02 | (5.5,6.0,2.0) |
| 9 | *T1* | 1.53 | (21.7,16.25,17.7) | 0.91 | (21.2,15.0,10.0) | 0.06 | (10.3,7,6.3) |
|  | *T2* | 1.3 | (20.7,15.2,17.5) | 0.90 | (21.2,15,10.1) | 0.07 | (10.5,7,6.2) |
| 10 | *T1* | 1.48 | (19.7,17,15.5) | 1.63 | (22.7,22,20.7) | 0.02 | (8.5,4.7,1.7) |
|  | *T2* | 1.5 | (20.7,16,16.5) | 1.6 | (22.5,22.1,20.5) | 0.03 | (10.5,4.5,2.3) |

**S1 Table. The source data, volume and all dimensions, for mandibular condyle, glenoid fossa and articular disc at time 1 (T1) and time 2 (T2).**
